# Does the genetic structure of spring snail *Bythinella* (Caenogastropoda, Truncatelloidea) in Bulgaria reflect geological history?

**DOI:** 10.3897/zookeys.518.10035

**Published:** 2015-08-25

**Authors:** Artur Osikowski, Dilian Georgiev, Sebastian Hofman, Andrzej Falniowski

**Affiliations:** 1 Department of Comparative Anatomy, Institute of Zoology, Jagiellonian University, Gronostajowa 9, 30–387, Kraków, Poland; 2 Department of Ecology and Environmental Conservation, University of Plovdiv, Tzar Assen Str. 24, BG-4000 Plovdiv, Bulgaria; 3 Department of Malacology, Institute of Zoology, Jagiellonian University, Gronostajowa 9, 30–387, Poland

**Keywords:** Gastropoda, phylogeography, Balkans, Messinian Salinity Crisis, Dacic Basin

## Abstract

*Bythinella* is a minute dioecious caenogastropod that inhabits springs in central and southern Europe. In the Balkans, previous studies have addressed its morphological and genetic differentiation within Greece and Romania while the Bulgarian species have remained poorly known. The aim of the present paper has been to expand the knowledge on the subject in Bulgaria. Shell morphology and anatomy of the reproductive organs were examined, and a fragment of the mitochondrial cytochrome oxidase subunit I (COI) gene and the nuclear ribosomal Internal Transcribed Spacer 1 (ITS-1) were sequenced from 15 populations. Additional sequences from eight previously studied populations were included in our analyses. Phylogenetic analyses revealed five main mitochondrial DNA clades, which were partly confirmed by analyses of the ITS-1 sequences. The genetic differentiation between the clades was found to be in the range *p*=2.4-11.8%. Most of the populations belonged to clade I, representing *Bythinella hansboetersi*, and were distributed in SW Bulgaria. Clades II and III inhabit areas adjacent to clade I and were most closely related with the latter clade. Much more distinct were clade V, found at one locality in NW Bulgaria, and clade IV, found at one locality in SE Bulgaria, close to the sea. Four populations were found in caves, but only one of these represented a distinct clade. Considering the observed pattern of interpopulation differentiation of *Bythinella* in Bulgaria, we can suppose that isolation between clades I, II and III may have been caused by glaciations during the Pleistocene. The time of isolation between the above three clades and clade IV coincides with the Messinian Salinity Crisis, and the time of isolation between the clade V and the other four most probably reflects the isolation of the Rhodopes from western Balkan Mts by the seawater of the Dacic Basin.

## Introduction

The genus *Bythinella* Moquin-Tandon, 1856 consists of minute (2–4 mm shell height), dioecious, oviparous freshwater snails inhabiting springs and subterranean waters across Europe and western Asia. In Europe, their range extends from the Iberian Peninsula to the Ukraine, and from southern Poland and Germany, to Sicily and Crete. These snails also occur on numerous Mediterranean islands and in the western part of Turkey (Falniowski et al. 2012; Benke et al. 2011).

Early studies on *Bythinella* taxonomy mainly concentrated on the morphology of the shell and, later, the external morphology and anatomy of the body (e.g., Boeters 1973, 1998; Radoman 1976; Falniowski 1987). It has been demonstrated, however, that morphology alone cannot be used for unequivocal species delimitation due to the limited number of taxonomically useful characters and their large variability (Falniowski 1987, 1992; Falniowski et al. 2009a; Bichain et al. 2007a, b). Recent molecular studies (Bichain et al. 2007a; Haase et al. 2007; Benke et al. 2011; Falniowski et al. 2009a, b, 2012; Falniowski and Szarowska 2011, 2012) have led to revised species delimitations. For *Bythinella* an allopatric mode of speciation and distribution of the species has generally been postulated. This caused authors to overestimate the importance and effectiveness of geographical isolation. This partly triggered numerous descriptions of new species, which were based solely on the occurrence of snails in springs not previously studied. However, the real amounts of gene flow between local populations of *Bythinella* is high (Falniowski et al. 1998, 1999, Falniowski and Szarowska 2011), and there have been even sympatric occurrences of two species of *Bythinella* in the same spring (Radoman 1976, Falniowski et al. 2009b, Falniowski and Szarowska 2011).

In Bulgaria, several *Bythinella* species have been described solely on the basis of morphological characters, including only shell and penis: *Bythinella ravnogorica*, Glöer and Georgiev 2009, *Bythinella rhodopensis*, Glöer and Georgiev 2009, *Bythinella srednogorica*, Glöer and Georgiev 2009, *Bythinella markovi*, Glöer and Georgiev 2009, *Bythinella walkeri*, Glöer and Georgiev 2009, (Glöer and Georgiev 2009), *Bythinella rilaensis*, Glöer and Georgiev 2011, *Bythinella slaveyae*, Glöer and Georgiev 2011, *Bythinella angelovi*, Glöer and Georgiev 2011 (Glöer and Georgiev 2011), Bythinella cf. opaca, von Gallenstein 1848 (Georgiev and Stoycheva 2008; later described as *Bythinella srednogorica*), *Bythinella gloeeri*, Georgiev 2009 (Georgiev 2009), *Bythinella hansboetersi*, Glöer and Pešić 2006 (Glöer and Pešić 2006) and *Bythinella stoychevae*, Georgiev 2011 (Georgiev 2011). In *Bythinella*, the shell as well as the penis exhibit substantial variability, often even within populations (Falniowski 1987), including all the character states considered by the authors cited above. Last but not least, the species-level taxonomy of *Bythinella* is heavily flawed by common assumption that different localities should harbour different species. Molecular studies by Falniowski et al. (2009a) on five populations from Bulgaria revealed low inter-population genetic distances, leading to the conclusion that all of them belonged to the same species. This result strongly suggests that it is necessary to incorporate the study of molecular markers in critical revisions of the *Bythinella* species in Bulgaria.

The palaeogeographic history of central and southern Europe has significantly influenced the distribution of fauna and flora in this region. An ecological event with a large impact on biodiversity in present day Bulgaria and Romania was the flooding of the Dacic Basin with seawater. This basin separated the Carpathians from central Bulgaria (Suc et al. 2011) from about 8 until about 1.8 Million years ago (Mya). Previous research has indicated that the genetic divergence of *Bythinella* is much higher in neighbouring Romania than in Bulgaria, suggesting that the Dacic Basin may have caused the extinction of this genus in a vast parts of Bulgaria (Falniowski et al. 2009b, 2012). However, this scenario was suggested based on limited sampling in Bulgaria and, therefore, requires further study.

Among many nominal species of *Bythinella* described in Bulgaria on the basis of morphological characters, some cave taxa have been identified. *Bythinella markovi* was reported from the Gargina Dupka Cave (Glöer and Georgiev 2009, 2011; Georgiev and Glöer 2013) and *Bythinella gloeeri* from Lepenitsa Cave in the Rhodopes (Georgiev 2009). These two caves are situated about 80 km apart from each other as the crow flies in different ridges of the Rhodopes as parts of different river catchments. These two species differ substantially in their morphology. Current phylogeographic studies confirmed theoretical assumptions that cave animal taxa are often cryptic and possess highly restricted geographical distributions despite potential gene flow from surface populations (Juan et al. 2010). To test this hypothesis on Bulgarian *Bythinella*, confirmation of its distinctness, previously described on basis of morphological characters, is necessary using molecular techniques. Since low genetic differentiation between the surface *Bythinella* population in Bulgaria has been reported (Falniowski et al. 2009a), more data on the phylogenetic differentiation of potentially distinct cave species is needed.

The aim of our study has been to improve the knowledge of *Bythinella* distribution in Bulgaria through extended sampling and to answer the following questions: 1) Is the low genetic divergence previously reported for Bulgarian *Bythinella* a fact or the result of poor sampling? 2) Are the biogeographical patterns and phylogenetic relationships of *Bythinella* correlated with the geological history of the region? 3) Have the cave populations of *Bythinella* been isolated for a long time from the ones in surface water reservoirs? 4) Do molecular data support the opinion of Glöer and Georgiev (2011) about Bulgaria as a hot-spot of diversity of *Bythinella*? To answer these questions, both morphological (shell) and molecular (COI and ITS-1 genetic markers) characters were examined.

## Materials and methods

### Snail sampling and fixation

**Figure 1.**
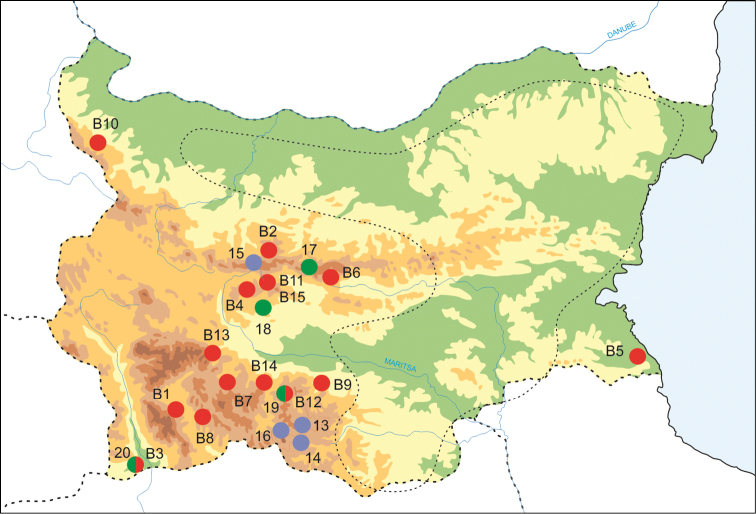
Sampling sites used in the present study (red dots) and in phylogenetic analyses (blue dots: Falniowski et al. 2009a; green dots: Falniowski et al 2012). Compare with Table 1. The dotted line indicates an area that was searched but where no *Bythinella* sites were found.

*Bythinella* snails were collected from 15 sites across Bulgaria (Fig. 1, Table 1). In four of these sites the snails were found in caves. Six of the studied populations were from the type localities of the nominal species.

**Table 1.** The sampling localities with their geographical coordinates, and the haplotypes for COI and ITS genes detected in each locality. Sequences from GenBank are also included. Compare with Figure 1.

ID	Taxon	Site	Coordinates	COI haplotypes	ITS haplotypes
B1	*Bythinella* sp.	Bulgaria - Bezbog Peak, Pirin Mts.	41°45'20"N; 23°32'39"E	HB1×3	HB1×3
B2	*Bythinella* sp.	Bulgaria - Ribarits village, Stara Planina Mts.	42°49'33"N; 24°22'23"E	HB2A×3, HB2B	HB2A,HB2B
B3	*Bythinella* sp.	Bulgaria - Leshnishki Waterfall, Belasitsa Mts.	41°22'12"N; 23°11'12"E	HB3A, HB3B×3	HB3
B4	*Bythinella* sp.	Bulgaria - Panagyurski Kolonii, Sredna Gora Mts.	42°34'20"N; 24°12'34"E	HB2A×3	HB2B×2
B5	*Bythinella* sp.	Bulgaria - Nestinarka beach, Strandzha Mts.	42°09'16"N; 27°51'21"E	HB5×6	-
B6	*Bythinella* sp.	Bulgaria - N of Pnitsite area, Stara Planina Mts.	42°39'44"N; 24°58'40"E	HB6×5	HB2A×2
B7	*Bythinella gloeeri*	Bulgaria - Lepenitza cave, West Rhodopes Mts.	41°57'14"N; 24°00'43"E	HB7A, HB7B×5	HB7×5
B8	*Bythinella stoychevae*	Bulgaria - Manuilova Dupka Cave, Rhodopes Mts.	41°42'53"N; 23°46'58"E	HB8×5	HB8×2
B9	*Bythinella ravnogorica*	Bulgaria - Ravnogor village, West Rhodopes Mts.	41°57'00"N; 24°21'53"E	HB9A, HB9B×2	-
B10	*Bythinella* sp.	Bulgaria - Vodni Pech Cave	43°30'11"N; 22°46'55"E	HB10A×2, HB10B, HB10C×3	HB10A×2, HB10B, HB10C×2
B11	*Bythinella angelovi*	Bulgaria - Koprivskitsa town, Sredna Gora Mts.	42°08'14"N; 24°21'55"E	HB11	HB2A
B12	*Bythinella rhodopensis*	Bulgaria - Modarskata Cave, West Rhodopes Mts.	41°52'40"N; 24°33'40"E	HB12×3	HB2A×2
B13	*Bythinella rilaensis*	Bulgaria - Rila Mts., near Belovo	42°08'15"N; 23°58'00"E	HB7B×2	HB7×4
B14	*Bythinella dierkingi*	Bulgaria - Ravnogor village, West Rhodopes Mts.	41°56'59"N; 24°22'02"E	HB11×3	HB2A×4
B15	*Bythinella* sp.	Bulgaria - Koprivshtitsa town, Sredna Gora Mts.	42°38'15"N; 24°21'57"E	HB11×4	HB2A×2, HB2B
Falniowski et al. 2009b (FJ545011–FJ545131)					
1	*Bythinella viseuiana*	Romania - Vişeu River Valley	47°52'14"N; 24°11'23"E	HR1A×3, HR1B×6	HR1×2
2	*Bythinella molcsanyi*	Romania - Igniş Mts., western slope of Firiza Lake	47°43'02"N; 23°36'29"E	HR2A×2, HR2B×5	HR2A, HR2B
3	*Bythinella molcsanyi*	Romania - Igniş Mts., upstream of locality 2	47°45'58"N; 23°38'32"E	HR2A×3, HR3A×5, HR3B×2	HR2B×2
4	*Bythinella molcsanyi*	Romania - Igniş Mts., near Izvoare Resort	47°45'14"N; 23°42'28"E	HR3A×2, HR4A×4, HR4B×3	HR4A×2, HR4B
5	*Bythinella molcsanyi*	Romania - Igniş Mts., Izvoare Resort	47°44'51"N; 23°43'03"E	HR2B×12, HR4B×8	HR5A, HR5B, HR5C,HR5D
6	*Bythinella radomani*	Romania - Bihor Mts., close to Vârtop Pass	46°31'25"N; 22°37'25"E	HR6A×5, HR6B×3	HR6A×2, HR6B, HR6C, HR6D
7	*Bythinella radomani*	Romania - Bihor Mts., Iarba Rea village	46°25'35"N; 22°46'29"E	HR7×7	HR7×3
8	*Bythinella dacica*	Romania - Retezat Mts., La Beci, Buta river valley	45°18'26"N; 22°56'12"E	HR8A×6, HR8B×4, HR8C×3	HR8A, HR8B
9	*Bythinella dacica*	Romania - Retezat Mts., Râu Şes valley	45°19'25"N; 22°40'51"E	HR9A×3, HR9B×4	HR9A, HR9B×2, HR9C
10	*Bythinella dacica*	Romania - Cerna Valley	45°00'33"N; 22°32'40"E	HR10A×2, HR10B×4	HR10A, HR10B, HR10C
11	*Bythinella dacica*	Romania - Cerna Valley, 3.5 km up from locality 10	45°02'10"N; 22°34'06"E	HR10A×6, HR11×6	HR10B×2
12	*Bythinella calimanica*	Romania - Cӑlimani Mts.	46°57'10"N; 25°04'07"E	HR12A×3, HR12B×4, HR12C×6	-
Falniowski et al. 2009a (GQ152518–GQ152544)					
13	*Bythinella hansboetersi*	Bulgaria - Smoljan town, below Smoljanske Lake	41°37'01"N; 24°40'31"E	HBU13A×2, HBU13B×2, HBU13C	HB2A, H13A, HBU13B
14	*Bythinella hansboetersi*	Bulgaria - Smoljan town, near Amzovo	41°33'42"N; 24°41'41"E	HB2A×2, HBU14A×3, HBU14B×2	HBU13B, HBU14
15	*Bythinella hansboetersi*	Bulgaria - Anton town, Bolovan Hill	42°44'48"N; 24°16'51"E	HB2A×3, HBU15A×3, HBU15B, HBU15C	HB2B×5, HBU15
16	*Bythinella hansboetersi*	Bulgaria - Mugla village	41°37'43"N; 24°31'08"E	HBU16A×4, HBU16B×3	HBU16A×2, HBU16B
Falniowski et al 2012 (JQ639859–JQ639883)					
17	*Bythinella hansboetersi*	Bulgaria - Stara Planina, spring of Cherni Osam	42°43'21"N; 24°46'47"E	HBU15B	-
18	*Bythinella srednogorica*	Bulgaria - Sredna Gora Mts., S. of Streltcha town	42°27'16"N; 24°20'27"E	HB2A×2, HBU18	-
19	*Bythinella rhodopensis*	Bulgaria - West Rhodopes Mts., S. of Lilkovo village	41°52'39"N; 24°33'21"E	HBU19A, HBU19B, HBU19C	-
20	*Bythinella slaveyae*	Bulgaria - Belasits Mts., S. of Belasitsa village	41°21'07"N; 23°09'19"E	HB2A×2, HBU20	-
21	*Bythinella nonveilleri*	Serbia - Rtanj Mt., Vrmd a spring	43°42'00"N; 21°49'00"E	HSE21A×2, HSE21B	-
22	*Bythinella pesterica*	Serbia - Pester Plateau, Djerekare village	43°00'00"N; 20°08'00"E	HSE22A, HSE22B, HSE22C	-
23	*Bythinella taraensis*	Montenegro - canyon of the river Tara, stream Ljevok	42°59'29"N; 19°25'53"E	HMO23A×2, HMO23B	-
24	*Bythinella luteola*	Montenegro - National Park Biogradska Gora	42°53'31"N; 19°36'16"E	HMO24	-
25	*Bythinella dispersa*	Montenegro - spring in Petnjik village	42°49'35"N; 19°54'10"E	HMO25A, HMO25B, HMO25C	-

Snails were collected by hand or with a sieve. Individuals for the morphological study were fixed in 4% formaldehyde and stored in 80% ethanol, while individuals for molecular analyses were washed in 80% ethanol and left to stand in it for about 12 hours. The ethanol was then changed twice during 24 hours and, after a few days, samples were transferred to 96% ethanol and stored at -20 °C prior to DNA extraction.

### DNA extraction and sequencing

DNA was extracted from foot tissue using the SHERLOCK extracting kit (A&A Biotechnology) and dissolved in 20 µl TE buffer. PCR was performed in the reaction mixture of 50 µl total volume using the following primers: LCOI490 (Folmer et al. 1994) and COR722b (Wilke and Davis 2000) for the COI gene, and two *Bythinella*-specific primers ITS1D and ITS1R for the ITS-1 (Bichain et al. 2007). The PCR conditions were as follows. COI – initial denaturation step of 4 min at 94 °C, followed by 35 cycles at 94 °C for of 1 min, 55 °C for 1 min, 72 °C for 2 min, and a final extension of 4 min at 72 °C; ITS-1 – initial denaturation step of 4 min at 94 °C, followed by 25 cycles at 94 °C for 30 s, 60 °C for 30 s, 72 °C for 30 s, and a final extension of 5 min at 72 °C. Ten µl of the PCR product was run on 1% agarose gel to check for quality. PCR products were purified using Clean-Up columns (A&A Biotechnology). The purified PCR products were sequenced in both directions using BigDye Terminator v3.1 (Applied Biosystems) following the manufacturer’s protocol and using the primers described above. The sequencing reaction products were purified using ExTerminator Columns (A&A Biotechnology), and the sequences were read using an ABI Prism sequencer.

### Morphological studies

Snails were dissected under a NIKON SMZ-U stereo-microscope with a NIKON drawing apparatus, and a CANON EOS 50D digital camera was used to photograph the shells.

### Data analysis

Sequences were edited in Bioedit 7.1.3.0 (Hall 1999) and aligned with the ClustalW program in MEGA 6 (Tamura et al. 2013). Single ITS sequences were assembled and aligned using CodonCodeAlligner 4.2.7 (CodonCodeCorporation, Dedham, MA). Basic sequence statistics, including haplotype polymorphism and nucleotide divergence, were calculated in DnaSP 5.10 (Librado and Rozas 2009). The saturation test of Xia et al. (2003) was performed using DAMBE (Xia 2013).

Sequences obtained from *Bythinella* specimens in the present work were used in a phylogenetic analysis with other sequences obtained from GenBank (Table 1). The data were analysed using Bayesian inference (BI) and the maximum likelihood (ML) approach. We applied the GTR + I + Γ model because over-parameterization seems to be less dangerous for BI analyses than under-parameterization (Huelsenbeck and Rannala 2004). For ML analyses, GTR + I + Γ is the only nucleotide substitution model implemented in RAxML.

The Bayesian analyses were run with MrBayes ver. 3.2.3 (Ronquist et al. 2012) using default priors. Two simultaneous analyses were performed, each lasting 10,000,000 generations, with one cold chain and three heated chains, starting from random trees and sampling trees every 1000 generations. The first 25% trees were discarded as burn-in. The analyses were summarised on a 50% majority-rule tree.

A maximum likelihood (ML) approach was conducted in RAxML v8.0.24 (Stamatakis 2014). One thousand searches were initiated with starting trees obtained through the randomized stepwise addition maximum parsimony method. The tree with the highest likelihood score was considered as the best representation of the phylogeny. Bootstrap support was calculated with 1000 replicates and summarized onto the best ML tree. RAxML analyses were performed using free computational resource CIPRES Science Gateway (Miller et al. 2010).

To infer haplotype networks of the markers used, a median-joining calculation was implemented in NETWORK 4.6.1.1 (Bandelt et al. 1999).

To test the molecular clock, COI data were used. Two hydrobiids, *Peringiaulvae* Pennant, 1777 and *Salenthydrobia ferreri* Wilke, 2003 (AF478401, AF478410) were used as outgroups. The divergence time between these two species (5.96 Mya) was used to calibrate the molecular clock, with correction according to Falniowski et al. (2008), since the isolation started with the beginning, not the end of the Messinian Salinity Crisis. The likelihoods for trees with and without the molecular clock assumption for a Likelihood Ratio Test (LRT) (Nei and Kumar 2000) were calculated with PAUP. The Relative Rate Test (RRT) (Tajima 1993) was performed in MEGA. As Tajima’s RRTs and the LRT test rejected the equal evolutionary rate throughout the tree for *Pseudamnicola*, time estimates were calculated using a non-parametric rate smoothing (NPRS) analysis with the recommended Powell algorithm, in r8s v.1.7 for Linux (Sanderson 1997, 2003).

## Results

**Figure 2.**
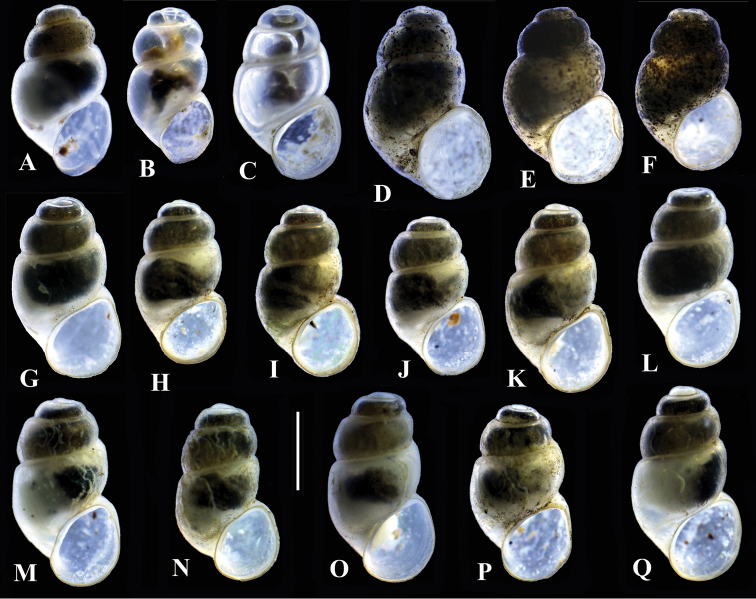
Shells of *Bythinella*. **A** locality B1 **B–C** locality B7 **D–F** locality B5 **G–Q** locality B10; bar equals 1 mm.

Selected shells of *Bythinella* from some of the studied localities are presented in Fig. 2A–Q. It is visible that the variability at one locality (B10: Fig. 2G–Q) is equivalent to the variation observed amongst all populations.

We obtained 58 new sequences of COI (552 bp, GenBank Accession numbers KT381098–KT381155) and 36 new sequences of ITS-1 (234–264 bp, GenBank Accession numbers KT381156–KT381191). For COI the saturation test of Xia et al. (2003) revealed no saturation. Seventeen COI haplotypes (haplotype diversity Hd = 0.932) and ten ITS-1 haplotypes (Hd = 0.837) were identified. For phylogenetic analyses, additional *Bythinella* sequences available in GenBank (Table 1) were included, including those from eight sites in Bulgaria (15 haplotypes: Falniowski et al. 2009a, 2012), twelve sites in Romania (22 haplotypes: Falniowski et al. 2009b) and five sites in Montenegro (six haplotypes) and Serbia (five haplotypes: Falniowski et al. 2012). The topologies of the resulting ML and BI phylograms were identical. Sequences of *Bythinella viridis* were used as outgroup in all analyses to root the trees.

**Figure 3.**
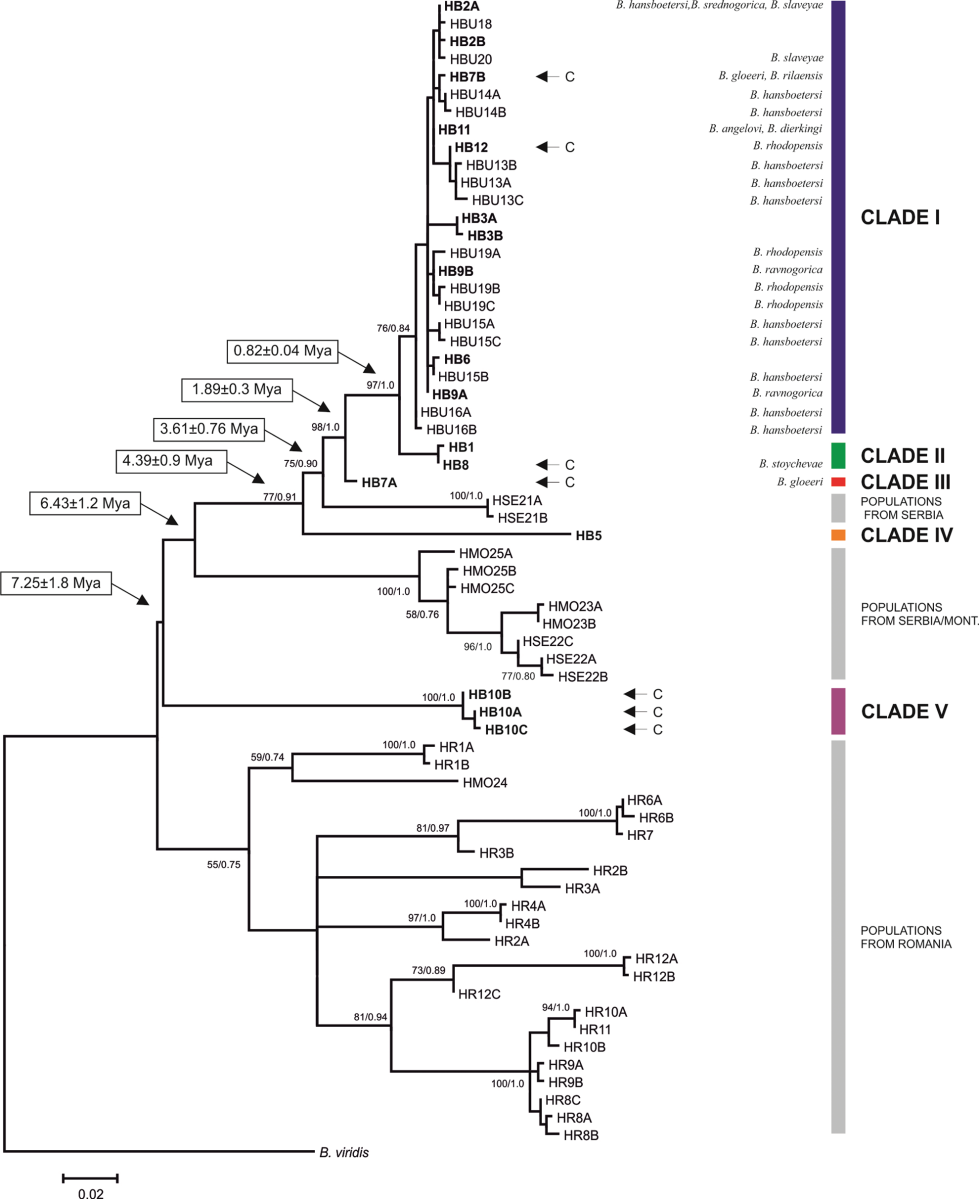
The maximum-likelihood phylogram for COI gene. Haplotypes obtained in present work are indicated in bold. Arrows and the letter C indicate cave haplotypes.

**Figure 4.**
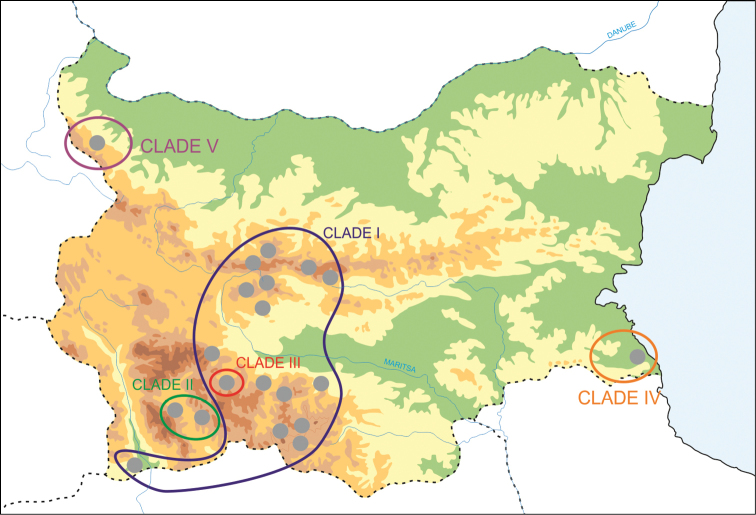
Geographical distribution of COI clades. Compare with Figure 3.

**Figure 5.**
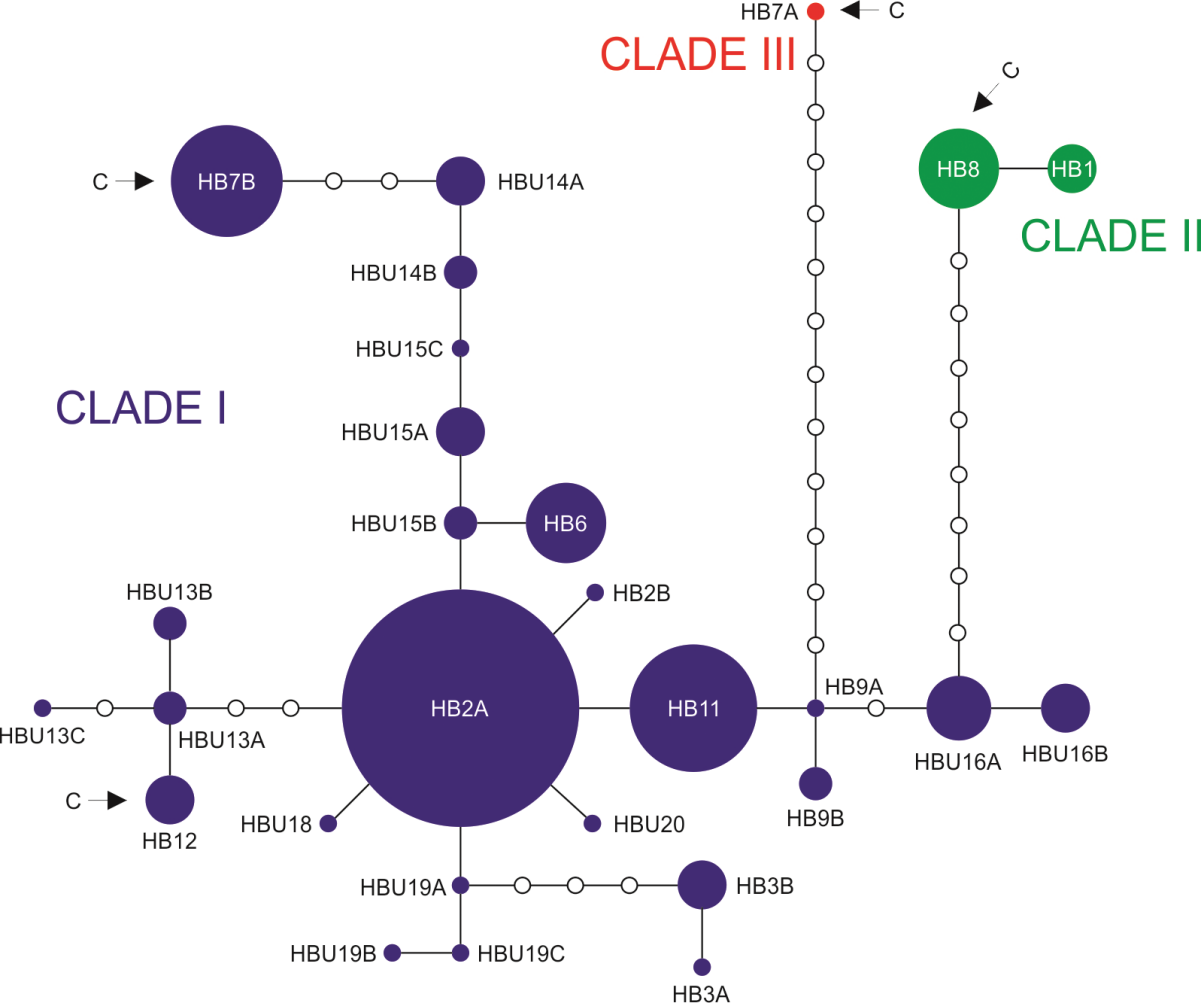
The median-joining haplotype network of COI haplotypes for clades I, II and III. Sequences from Falniowski et al. 2009a and Falniowski et al. 2012 are also included. Arrows and the letter C indicate cave haplotypes.

In the COI trees five main clades could be distinguished for the Bulgarian populations (Figs 3–4). Clade I included the largest number of haplotypes covering an area from the Rhodopes Mts through the Maritsa Valley to the Stara Planina and Sredna Gora Mts. This clade is characterized by a low sequence divergence (*p*-distance within this group = 0.008, Table 2). The relationships between the haplotypes of this clade are depicted in a haplotype network in Fig. 5. Most haplotypes from this clade belonged to snails inhabiting surface waters while two of them represent cave populations (Fig. 3). This clade represents several nominal species: *Bythinella angelovi*, *Bythinella dierkingi*, *Bythinella gloeeri*, *Bythinella hansboetersi*, *Bythinella ravnogorica*, *Bythinella rhodopensis*, *Bythinella rilaensis*, *Bythinella slaveyae*, and *Bythinella srednogorica*, in fact based mostly on their locations.

**Table 2.** Mean distances within clades (italics) and *p*-distances between main COI clades of *Bythinella*.

	clade_I	clade_II	clade_III	Serbia	clade_IV	Serbia/Mont.	clade_V	Romania
	*0.008*							
clade_II	0.024	*0.002*						
clade_III	0.026	0.031	-					
Serbia	0.063	0.070	0.054	*0.002*				
clade_IV	0.089	0.090	0.078	0.089	-			
Serbia/Mont.	0.109	0.112	0.096	0.119	0.122	*0.023*		
clade_V	0.091	0.090	0.084	0.090	0.118	0.126	*0.004*	
Romania	0.112	0.106	0.097	0.120	0.122	0.118	0.109	*0.079*

Clades II and III were most closely related to Clade I differing by intercladal *p*-distances of 0.024 and 0.026 and inferred divergence times of 0.82 and 1.89 Mya, respectively (Table 2). Clade II contained haplotypes from two sites in the south-west of Bulgaria: the Pirin Mts and a cave in the Rhodopes Mts, from which *Bythinella stoychevae* has been described. Only one haplotype formed Clade III representing *Bythinella gloeeri*. All other sequences of this nominal species belonged to Clade I, however.

The haplotype from the easternmost site (B5, in the Strandzha Mts) formed Clade IV. It differed from clades I to III by genetic distances of 7.8 to 9.0% (inferred divergence time 4.39 Mya) (Table 2). This clade is situated between the two reference clades from Serbia and Montenegro. The most divergent clade was Clade V (inferred divergence time 7.25 Mya), formed by three haplotypes from the Vodni Pech Cave in north-western Bulgaria.

The reference sequences formed three distinct clades (Fig. 3). First of them represent one population from eastern Serbia, second the populations from western Serbia and Montenegro. Haplotypes from Romania formed another distinct lineage. The level of divergence between haplotypes within this lineage has been much larger than within the other clades.

**Figure 6.**
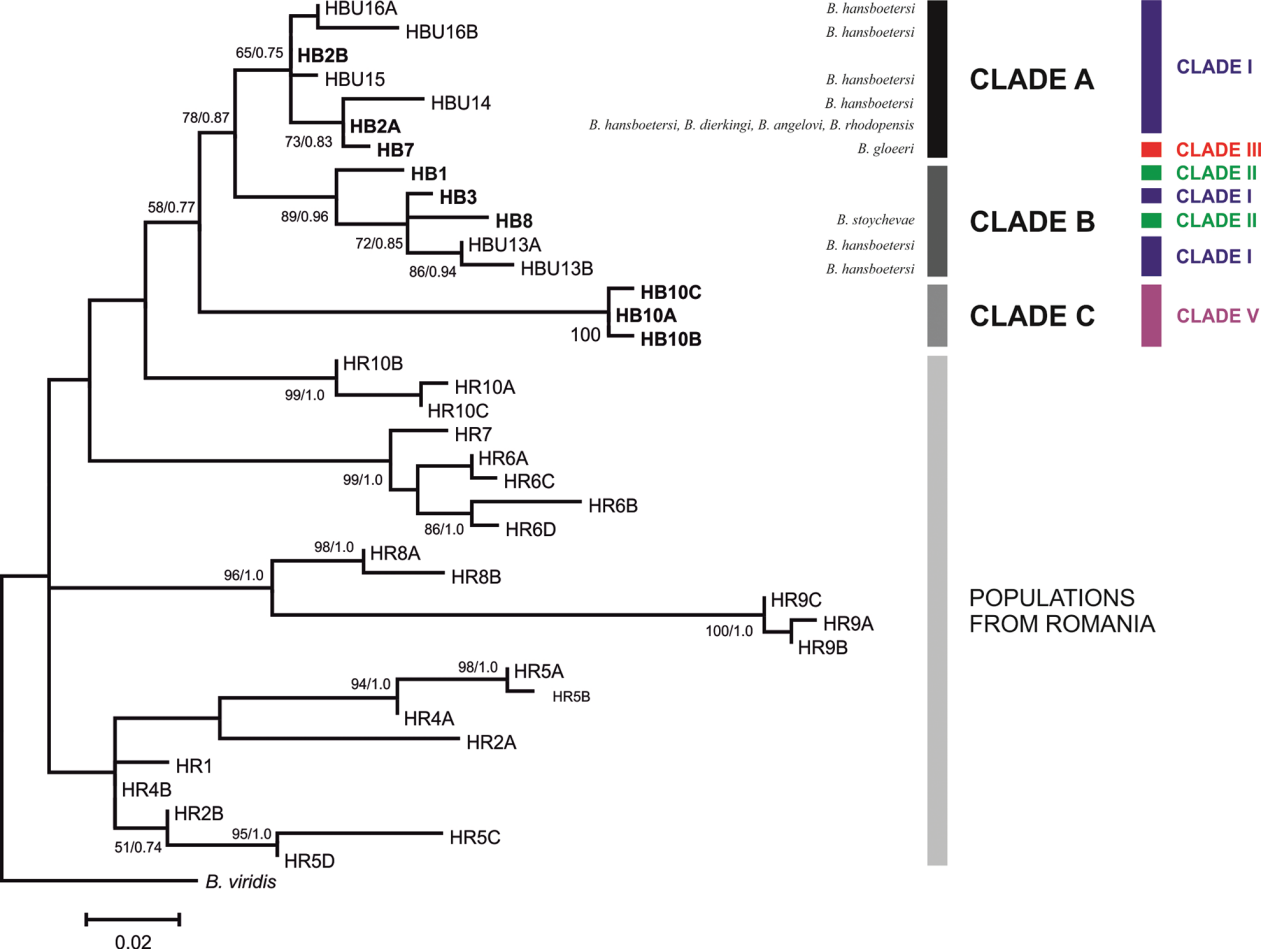
The maximum-likelihood phylogram for the ITS-1 gene. Haplotypes obtained in present work are shown in bold. The COI clades are also shown.

Unfortunately, due to technical problems, ITS-1 sequences were not available for samples from Clade IV and for the reference populations from Serbia and Montenegro. The ITS-1 tree (Fig. 6) confirmed the distinctiveness of the Bulgarian *Bythinella* from the Romanian ones. Three clades (A, B, C) could be distinguished. In correspondence with the COI tree, Clade C, containing sequences from the population from the Vodni Pech Cave, was found to be the most divergent (similarly as COI haplotypes from this population). Clade A comprised about half of all haplotypes of COI Clade I plus the COI Clade III. Clade B comprised all samples from COI Clade II and the rest of the samples from COI Clade I.

Shells from different clades (Fig. 2) were found to differ in morphology, but no clade-specific shell characters were found. Similar remarks concern both female reproductive organs, and the penes: no clade-specific character states were found.

## Discussion

Species delimitation in the genus *Bythinella* remains unclear. New species descriptions were initially based mainly on shell morphology, and on the locality not studied so far. Even for Radoman (1976), whose experience and extensive studies on the truncatelloidean anatomy were a basis for the new taxonomy proposed by him, the shell characters alone were the only basis for species-level taxonomy. He even stated that there could not be any differences in soft part morphology and anatomy between the congeneric species. Later, anatomy, especially of the reproductive system was considered (Boeters 1973, 1998; Falniowski 1987). In many cases, the number of species recognized from different parts in Europe is probably overestimated and the characters traditionally used to delimit species should be re-evaluated (Bichain et al. 2007b). In general, in *Bythinella* it is impossible to distinguish particular species without molecular data (Szarowska and Falniowski 2008; Falniowski et al. 2009b, c; Falniowski and Szarowska 2011). Analysis of the COI genetic distances between recognized clades/lineages could be considered an efficient tool for the rapid assessment of biodiversity in *Bythinella* (Bichain et al. 2007a).

Bichain et al. (2007a), after COI analysis, proposed a *K*2*P* value of 1.5% as the species threshold for European *Bythinella*. However, such a threshold value may be biased as well. So delimitation of *Bythinella* species needs to be backed up by additional data (e.g., more nuclear genes and mtDNA fragments, as well as detailed morphological studies). The history of any DNA fragment not necessarily reflects the history of speciation (Avise 2000), so multilocus analyses are necessary. The five molecularly distinct clades we have found may represent five distinct species. Especially the amount of mitochondrial differentiation of the COI clade V: 8.4–12.6 %, is within the range characteristic of the species level, and this divergence was confirmed by the ITS-1 as well. As could be clearly seen in the trees (Figs 3 and 6), there is rampant incongruence between phylogenetic patterns and current species-level taxonomy. Only *Bythinella gloeeri* (Clade III) and *Bythinella stoychevae* (Clade II) have been found to be molecularly distinct from *Bythinella hansboetersi* (Clade I). However, all morphological character states given in the descriptions of the Bulgarian species are variable even within a population in *Bythinella* (Falniowski 1987, Mazan 2000, Mazan and Szarowska 2000a, b).

Most populations examined here occurred in the area from the Rhodopes Mts to the Sredna Gora Mts (Clades I, II, III). Only two other isolated populations were found in eastern Bulgaria, in the Strandzha Mts (Clade IV) and in the north-western part of this country (Clade V). Despite an extensive search, no members of this genus were found in the rest of Bulgaria (Fig. 1). However, there are several areas where no *Bythinella* occur, since these snails are sensitive to environmental conditions, such as high water calcium content and low temperature. They occasionally occur in spring outlets and creeks, and also in caves or groundwaters (Giusti and Pezzoli 1977, 1980). Water conditions may be one of the most important factors influencing the occurrence of *Bythinella*. On the one hand, regional and global environmental changes may have relatively small effects on this spring snail, since springs can buffer such changes. On the other hand, *Bythinella* is more resistant than had been expected for a long time (e.g., Szarowska 2000) and, since springs are ephemeral habitats that are certainly not long-lasting, there must be unexpectedly high gene flow between them to colonize/recolonize them (e.g., Falniowski et al. 1998, 1999).

Low, infraspecies-level diversity characterized Clade I, including most of the studied populations distributed across central and western Bulgaria. The representatives of Clades II, III and V either migrated from the west, or survived there from earlier time. It seems possible that both clades III and V survived glaciations inside the caves. Clade IV most probably originated in the present Asia Minor. The closest sequences to clade V come from the *Bythinella turca* haplotype from the Egirdir Lake in Turkey (*p* distance = 0.055).

Mitochondrial interpopulation differentiation of Bulgarian *Bythinella* (*p* distance = 0.03) is much smaller than in neighbouring countries (*p* distance = 0.06-0.08). The greater genetic differences between *Bythinella* populations in Romania have been compared with the surprisingly low differentiation amongst the Bulgarian ones by Falniowski et al. (2009a,b). However, this analysis was only based on a small number of Bulgarian populations. The more detailed sampling in the present work confirmed this phenomenon. Moreover, lower interpopulation differentiation than in the Romanian *Bythinella* was also demonstrated for Greek populations (Falniowski et al. 2011) and throughout the East Balkans (Falniowski et al. 2012).

Within Bulgaria, some geological events could explain the low divergence in Clade I. The Dacic Basin, a vast water body that separated the Carpathians from the recent central Bulgaria before and just after the peak of Messinian Salinity Crisis (5.60–5.46 Mya) (Popov et al. 2004, 2006; Popescu et al. 2009; Suc et al. 2011), was a part of the Paratethys, connected with the Pannonian Basin in the west, the Euxinian Basin in the east, and directly with the present Aegean Sea in the south. Although its water-filled area eventually decreased in size, it was still present until the middle Pleistocene, about 1.8 Mya. The Dacic Basin most probably separated the ancestors of the two large clades, about 8 Mya. Later, in the Pleistocene, the unstable fluviolacustrine system in south-western Bulgaria and northern Greece, with glaciers present in the Pirin and Rila Mts (Zagorchev 2007), probably formed effective, temporary barriers for *Bythinella*, and may have caused its extinction in most of Bulgaria. Considering the data known so far, the small differences among the Bulgarian populations representing Clade I may reflect the short history of *Bythinella* in the area, which was most probably recolonised from the south, certainly not from the north, no earlier than in the late Pleistocene.

Benke et al. (2011) revealed that genetic diversity of *Bythinella* in Europe is not distributed equally, and identified five “hotspots”: Massif Central and Pyrenees, western Alps and northern Apennines, eastern Alps, western Carpathians and eastern Carpathians. The authors of the present paper discovered another *Bythinella* hotspot in central Greece (Szarowska et al. 2015). Thus, all the hotspots occur in mountain areas, which strongly suggests, that this type of landscape is especially favourable for *Bythinella*.

Moreover, almost all these hotspots are in places that were previously identified as *Bythinella* Pleistocene glacial refugia (Benke et al. 2009; Falniowski et al. 2009b), so high differentiation level in Romania may be the result of glaciations. During the Pleistocene these areas were probably a set of small areas of a nunatak character, with a mild climate suitable for *Bythinella* survival (Falniowski et al. 2009). Habitat fragmentation and subsequent periods of isolation in such shelters must have promoted speciation and could explain high differentiation level. It is widely accepted that, during glacial periods, the Pontic-Mediterranean refugium included territory in present-day Romania (e.g., Falniowski et al. 2009b). It seems that there is no trace of such refugium in Bulgaria.

Caves are relatively stable long-lasting environments and individual ones often have an island character with no subterranean connections to any others. In some cases, particular caves can be characterized by endemic taxa with long, independent, evolutionary histories (e.g., Falniowski et al. 2008, Juan et al. 2010 for references) that differ strongly from their sister taxa occurring outside caves. *Bythinella* inhabits both surface and underground waters, providing thus opportunity to compare populations from those two kind of habitats. In Bulgaria, only clade V was formed by haplotypes clearly distinct from the remaining *Bythinella* populations, which may reflect their troglobiontic character, and longevity of isolation, approaching the Pliocene. The collected individuals were, in fact, found not inside the cave, but at its entrance, in the water running from the cave, but this is normal way of collecting of several troglobiontic gastropods. In this population three haplotypes were found, in COI as well as in ITS-1, which is not common in troglobiontic animals (Juan et al. 2010), whose populations are usually monomorphic. This polymorphism may also confirm the longevity of this population, or inhabiting the cave by more than one species. Clade III was formed by a single COI haplotype from Lepenitza cave, locality B7, and represents *Bythinella gloeeri*. This haplotype, originated probably in the Calabrian, Pleistocene, also presents a distinct, probably troglobiont lineage. In both cases – COI clades V and III, the “climatic relict” hypothesis, as proposed by Howarth 1973 (Holsinger 1988, 2000; Peck and Finston 1993, Rivera et al. 2002). Accordin to this hypothesis, after the colonization of subterranean habitats there still takes place gene flow between the subterranean and surface population, but later, in strict allopatry, the subterranean population still speciates, and the surface population becomes extinct as a result of climatic changes, like glaciations or growing aridity. This seems a typical pattern for temperate climate. It has to noted, however, that at the same cave there was found another haplotype, belonging to the COI clade I. This is one more example of sympatric occurrence of more than one *Bythinella*. Such a situation is not unlikely and has been previously reported for *Bythinella* (Radoman 1976; Falniowski et al. 2009b, Falniowski and Szarowska 2011). This haplotype is close to the one inhabiting surface waters at locality B14, situated close to this cave. Similarly, the COI haplotype from troglobiont population at locality B12 (Modarskata Cave) was close to the one from the surface population 13, situated closely to population B12; the same concerns the troglobiont population B8 from Manuilova Dupka Cave compared with surface population B1. Low divergence between those troglobiont populations and their surface relatives may reflect either the early phase of the “climatic relict”-model processes, or “adaptive shift”-model: adaptive evolution of the lineages invading subterranean habitats, coupled with survival of the ancestral population at the surface.

Considering the observed pattern of interpopulation differentiation of *Bythinella* in Bulgaria, the facts listed above, and the divergence time estimates, we could suppose that the isolation between clades I, II and III (0.82 Mya and 1.89 Mya, respectively) may have been caused by subsequent glaciations during the Pleistocene. The time of isolation between the above three clades and clade IV from SE Bulgaria (4.39 Mya) coincides with the Messinian Salinity Crisis. Later, the low level of the present Black Sea promoted migration of the representatives of this clade from Asia Minor to Europe. The distinctness of clade V, found at NW Bulgaria, most probably reflects the isolation of the Rhodopes from the western Balkan Mts by the Dacic Basin (7.25–1.8 Mya).
